# Hormonal Influence on Coenzyme Q_10_ Levels in Blood Plasma

**DOI:** 10.3390/ijms12129216

**Published:** 2011-12-09

**Authors:** Antonio Mancini, Roberto Festa, Sebastiano Raimondo, Alfredo Pontecorvi, Gian Paolo Littarru

**Affiliations:** 1 Department of Internal Medicine, Division of Endocrinology, Catholic University of the Sacred Heart, Largo Gemelli, 1-00168 Rome, Italy; E-Mails: bastio984@hotmail.com (S.R.); a.pontecorvi@rm.unicatt.it (A.P.); 2 Department of Molecular and Clinical Sciences, Polytechnic University of Marche, Via Tronto 10, A-60020, Ancona, Italy; E-Mail: festa7r@libero.it; 3 Department of Biology, Biochemistry and Genetics, Polytechnic University of Marche, Via Ranieri, Montedago, 60128, Ancona, Italy; E-Mail: g.littarru@univpm.it

**Keywords:** coenzyme Q_10_, thyroid, oxidative stress, antioxidants, pituitary

## Abstract

Coenzyme Q_10_ (CoQ_10_), also known as ubiquinone for its presence in all body cells, is an essential part of the cell energy-producing system. However, it is also a powerful lipophilic antioxidant protecting lipoproteins and cell membranes. Due to these two actions, CoQ_10_ is commonly used in clinical practice in chronic heart failure, male infertility, and neurodegenerative disease. However, it is also taken as an anti-aging substance by healthy people aiming for long-term neuroprotection and by sportsmen to improve endurance. Many hormones are known to be involved in body energy regulation, in terms of production, consumption and dissipation, and their influence on CoQ_10_ body content or blood values may represent an important pathophysiological mechanism. We summarize the main findings of the literature about the link between hormonal systems and circulating CoQ_10_ levels. In particular the role of thyroid hormones, directly involved in the regulation of energy homeostasis, is discussed. There is also a link with gonadal and adrenal hormones, partially due to the common biosynthetic pathway with CoQ_10_, but also to the increased oxidative stress found in hypogonadism and hypoadrenalism.

## 1. Introduction

**Figure 1:**
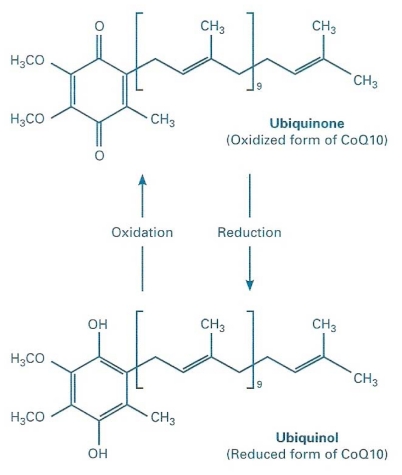
Chemical structure of Coenzyme Q_10_, in its two redox forms.

Coenzyme Q_10_ (CoQ_10_) is the predominant form of ubiquinone in man. The name ubiquinone (or ubidecarenone) refers to its ubiquitous diffusion in organisms and tissues. It is a key component of the ATP-producing oxidative phosphorylation chain, placed in the inner mitochondrial membrane, and linking flavoproteins and cytochroms. However, it is also a powerful lipophilic antioxidant, in particular in its reduced form, ubiquinol (Figure 1), which represents more than 80% of the total CoQ_10_ in human plasma and protects biological membranes and lipoproteins. Furthermore, new roles in different cellular functions have been discovered recently, regarding other cell organelles like lysosomes, Golgi apparatus and plasmatic membranes [1]. Finally, CoQ_10_ can participate in many aspects of the oxido-reductive control of the cellular signaling pathways, at least via the auto-oxidation of semi-quinon which can be a primary source of H_2_O_2_ generation [2]. Also CoQ_10_ involvement in cell proliferation was investigated [1], but most data at present concerns its energetic and antioxidant roles [3]. CoQ_10_ levels in biological fluids can be accurately measured by HPLC, with a method standardized by the International CoQ_10_ Association. Furthermore, electrochemical detection (HPLC-EC) allows measuring both the reduced (ubiquinol) and oxidized (ubiquinone) forms of CoQ_10_ [4]. The CoQ_10_ normal value range in human plasma is 0.8–1.2 mg/L. Since CoQ_10_ in the blood stream is prevalently transported in lipoproteins and above all low-density lipoproteins (LDL), CoQ_10_ values are commonly normalized for those of cholesterol (nmol/mmol), in order to minimize the confounding effect of cholesterol. Systemic hormones are variously involved in the regulation of metabolism, and act as a certain modulation on body antioxidant systems, both in physiological and pathological conditions, with hormone excess or deficiency [5]. The aim of this paper is to summarize the main findings of the literature about the link between hormonal systems and circulating CoQ_10_ levels.

## 2. CoQ_10_ Functions

### 2.1. Bioenergetical Role of CoQ_10_

**Figure 2:**
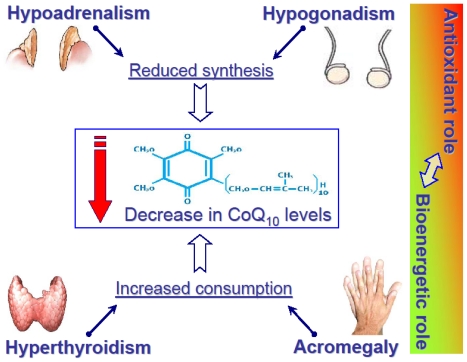
Alteration of CoQ_10_ levels in four “paradigmatic” endocrine disorders. The column on the right side refers to the pathophysiological phenomenon that if CoQ_10_ is involved largely as an antioxidant, then its bioenergetical role may be impaired, and *vice versa*.

CoQ_10_ was initially isolated as a yellow compound in 1957, from beef heart mitochondria by the group of Dr. D. Green at the University of Wisconsin. Then, Dr. K. Folkers determined its structure at the research laboratories of Merck in New Jersey; it is a moderately large worm shaped lipid molecule about 4 nm long, which can be described as: 2,3-dimethoxy-5-methyl-6-decaprenil-1,4-benzoquinone. CoQ_10_ was soon recognized as essential for the bioenergetics of cell respiration and named a coenzyme because of its activity in the enzyme systems of mitochondria. The letter Q indicates its quinonic group, while 10 are the isoprenoid units in its sidechain. CoQ_10_ carries hydrogens through the respiratory chain catalytic centres in the mitochondrial christae membrane, allowing coupling of the translocation of electrons to the translocation of protons, needed for the formation of chemiosmotic gradient for the functioning of ATP synthetase. Since CoQ_10_ affinity for the enzymes it interacts with, is not high enough to saturate them at its physiological concentration in the membrane, then the velocity of respiratory chain is very sensitive to variations of total CoQ_10_ or ubiquinole/ubiquinone ratio [6]. This concept links together the classical bioenergetic role of CoQ_10_ and its antioxidant role: any condition of increased oxidative stress, with the involvement of CoQ_10_ as antioxidant, might decrease its availability for oxidative phosphorylation (Figure 2). This is also the rationale for the clinical use of exogenous CoQ_10_ in different conditions and diseases.

### 2.2. CoQ_10_ and Oxidative Stress

Oxidative stress is defined as the unbalance between production of free radicals, molecules characterized by high chemical reactivity, and antioxidant defenses, in the biological systems. Oxidative stress is considered an important basic pathogenetic mechanism in different diseases. CoQ_10_ is an important mechanism of defense against oxidative stress. In fact, the most important and studied free radicals are reactive oxygen species (ROS), normally produced during oxidative processes of energetic substrates in the mitochondrial respiratory chain [7,8]. An increase in ROS production can be due to an increase in the electronic flow in the respiratory chain, resulting from an augmented energetic demand or an augmented disposal of substrates [9]. In leukocytes as well as many other cytotypes (endothelial and mesangial cells, fibroblasts, thyreocytes, oocytes, Leydig cells, adipocytes, Epstein-Barr and neoplastic cells) ROS generation was shown to have a positive pathophysiologic role, different from respiratory burst [10]. However an uncontrolled production of free radicals was linked to many pathologic events, such as rheumatoid arthritis and myocardial infarction, and in general ROS damage occurs in inflamed tissues, characterized from cellular lysis and intracellular content release. Moreover, in diabetes mellitus, oxidation, accompanying non-enzymatic glycation, supports the formation of irreversible chemical modifications on proteins and other kinds of molecules. The formation of these glycoxidation products depends not only on the relative glucose concentrations, but also on the local oxidative environment. On the other hand, in diabetic patients antioxidant capacity is decreased, finally resulting in an increased susceptibility to oxidative stress [11].

It is possible to characterize different cellular defensive mechanisms against the free radical damage, which act in the endoplasmic network, mitochondria, plasmatic membrane, peroxisomes and cytosol, as well as extracellular ambient. The first mechanism is the prevention of production or the rapid inactivation of free radicals, thanks to the action of enzymes, like catalase, peroxidase glutathion complex and superoxydedismutase (SOD), or of transition-metals binding proteins, like transferrin, ferritin and ceruloplasmin. The second mechanism interrupts propagation of the lipid peroxidation chain by inactivating the intermediate radicals. This mechanism is carried out by molecules called “scavengers”, which can be water-soluble, such as albumin, bilirubin, ascorbic acid, urates and thiols, or liposoluble, e.g., vitamin E and CoQ_10_, the only liposoluble antioxidant synthesized in living organisms. The mobility of scavengers, particularly the liposoluble ones and, most especially at the membrane level, allows interception of radicals and transforms them into more stable molecules and therefore stops damaging the chain. Sometimes scavengers can be regenerated, that is the case of CoQ_10_. The third defensive mechanism uses processes which remove molecules damaged by oxidative attack, allowing the reconstitution of normal structures; for instance, specific phospholypases remove the peroxidized fatty acids, enabling the re-acylation of damaged molecules by an acyl-CoA and the respective enzyme [12].

## 3. Clinical Significance of CoQ_10_ Measurement

The clinical value of CoQ_10_ is clear with respect to the antioxidant protection of lipoproteins. In fact, circulating LDLs are particularly prone to oxidative damage with generation of cytotoxic products, associated with atherosclerosis [13]. In LDLs, CoQ_10_ is oxidized before vitamin E, and the appearance of fat acids hydroperoxides occurs only after ubiquinol depletion [14], indicating CoQ_10_ as a first-line barrier against oxidative stress. Moreover, the treatment *per os* with exogenous CoQ_10_ in normal subjects induces an increase of ubiquinol levels in plasma and lipoproteins and an augmented resistance to LDL peroxidation [15]. Oxidative stress, obesity, metabolic syndrome and insulin resistance are crucial elements in the pathogenesis of atherosclerosis and cardiovascular disease, because of the association with lipoproteins rich in triglycerides (small and dense LDLs), oxidized LDLs, antibodies anti-oxidized LDLs and other oxidized and glycated particles, F2-isoprostans, soluble adhesion molecules, augmented levels of fibrinogen and PAI-1 e low levels of t-PA, an increase in CRP, IL-6, AA, omocistein, advanced glycation products [16–18]. Dealing with diabetes, blood plasma and cellular antioxidant defense is often reduced [19], and also CoQ_10_ plasma levels were found to be decreased in diabetic patients [20]. So we understand why lipoproteins isolated by diabetic subjects are more susceptible to the oxidation process.

The significance of oxidative stress in coronary cardiopathy has been investigated in a case-control study [21] and in two important cohort studies, the “Nurses’ Health Study” [22] and the “Physicians’ Health Study” [23]. The positive effect of the treatment with CoQ10 on angina pectoris, total arrhythmias, and left ventricular function after myocardial infarction was shown in the short term [24], as well as the long-term prognosis [25]. In conclusion, there is a relationship between low concentrations of plasma CoQ_10_ and coronary disease, even if this correlation is not so strong as even to be considered a casual relation [26]. However, ubiquinol/ubiquinone ratio is considered an oxidative stress marker in coronary disease and LDL/CoQ_10_ ratio was proposed as an index of coronary risk factor [20].

There are a lot of medical investigations concerning the potential therapeutic usefulness of CoQ_10_ in the treatment of various diseases, including endocrine ones: cardiovascular, neurological, muscular, immunologic, dental, diabetes, male infertility [3,27].

## 4. CoQ_10_ and Thyroid

Both hyperthyroidism and hypothyroidism are associated with enhanced oxidative stress involving enzymatic and non-enzymatic antioxidants [28]. In particular, hyperthyroidism is associated with reduced circulating levels of α-tocopherol [29,30] and CoQ_10_ [31,32], and some complications of hyperthyroidism are due to oxidative stress in target tissues [33]. Increasing CoQ_10_ values were found when going from hyper- to hypo-thyroidism, with euthyroism in the middle [34], and in a previous study we showed a significant inverse correlation between thyroid hormones and plasma CoQ_10_ [35]. This correlation was further confirmed in other studies and in a larger group of patients [32], and could cover diagnostic usefulness in those clinical conditions characterized by uncoupling of thyroid hormone levels and metabolic status, like amiodarone-induced thyroid dysfunction [35] and inappropriate thyroid stimulating hormone secretion.

Values of CoQ_10_ in hyperthyroid patients are among the lowest reported in different human diseases (Table 1). The reasons for this phenomenon include: decreased synthesis related to competition for tyrosine, which is a common substrate for CoQ_10_ and thyroxin synthesis (but this hypothesis was disconfirmed by experimental data in animals; increased CoQ_10_ utilization, due to the increased metabolic demand; increased degradation; decreased levels of carriers in serum, since it is demonstrated that release of very-low-density lipoproteins from liver is decreased in hyperthyroidism. Symmetrical mechanisms can be invoked to explain high CoQ_10_ levels in hypothyroid patients.

**Figure 3:**
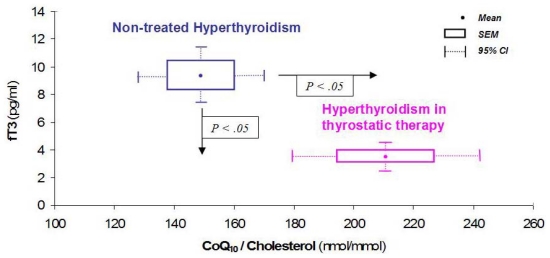
Effect of thyrostatic therapy on CoQ_10_ levels. Adapted with permission from [5].

In patients who underwent total thyroidectomy for papillary carcinoma or multinodular goiter, we found that patients with non toxic multinodular goiter exhibited low CoQ_10_ values in pathological areas, probably related to colloid accumulation; on the contrary, in active proliferating tissues (toxic goiter or neoplasm) CoQ_10_ concentrations were greater than in unaffected areas [32]. These data pointed toward increased CoQ_10_ uptake related to increased metabolic demand. Pharmacological treatment with metimazole, restoring normal values of thyroid hormones, also normalizes CoQ_10_ values (Figure 3), as seen in hyperthyroidal children too, even after CoQ_10_ was adjusted to cholesterol concentration, confirming a lipid-independent effect (apart from a decreased carrier capacity in serum) of the hyperthyroid state on the CoQ_10_ levels [5,36].

More recently, we tested a group of patients with low-T_3_ syndrome due to chronic obstructive pulmonary disease: again CoQ_10_ was higher than patients with the same disease and normal T_3_ levels, suggesting that real hypothyroidism could be present in such situation [37].

## 5. CoQ_10_ in Other Endocrine Diseases

### 5.1. Adrenal Disease

Due to the importance of oxidative stress in the pathophysiology of adrenal gland, we performed studies evaluating both blood plasma Total antioxidant capacity (TAC) and CoQ_10_. A further rationale for studying CoQ_10_ in pituitary-adrenal disease was the common biosynthetic pathway of cholesterol and ubiquinone. CoQ_10_ levels were significantly lower in isolated hypoadrenalism than in patients with adrenal hyperplasia and multiple pituitary deficiencies [38]. These preliminary data indicate that secretion of adrenal hormones is in some way related to CoQ_10_ levels, both in augmented and reduced conditions. However in secondary hypoadrenalism, some other pituitary dependent axes can be affected. Therefore we compared patients with post-surgical isolated hypoadrenalism with those who also presented hypothyroidism. Since thyroid hormones play an important role in modulating CoQ_10_ levels and metabolism, when coexistent (Table 1), thyroid deficiency seems to play a prevalent role instead of adrenal deficiency [39].

### 5.2. Gonadal Disease

To investigate the role of gonadal steroids in systemic antioxidant regulation, we determined plasma CoQ_10_ and its contribution to TAC in post-surgical hypopituitaric patients. Sixteen out of 26 patients presented low testosterone values and were also studied after treatment with testosterone enantate. CoQ_10_ levels were significantly lower in isolated hypogonadism than in normogonadism. Testosterone treatment induced a significant change both in CoQ_10_ level and TAC. CoQ_10_ and TAC values significantly correlated, suggesting an inter-relationship between different antioxidants [40].

Our data suggest that hypogonadism could represent a condition of oxidative stress, in turn related with augmented cardiovascular risk. Once again, when hypogonadism was associated to hypothyroidism, the effect of the latter was prevalent (Table 1).

### 5.3. Growth Hormone

A previous study of our group also evaluated acromegalic patients [41], harboring a GH-secreting pituitary adenoma. In this case, we observed lower CoQ_10_ values; however sometimes the pituitary adenoma can damage other pituitary cell lines or disrupt the connection with hypothalamus, causing defects in other pituitary dependent axes (thyroid, adrenal, gonadal). In the case of secondary hypothyroidism, as seen above, this last phenomenon can be prevalent; therefore in acromegalic patients with concomitant hypothyroidism, CoQ_10_ plasma levels are increased (Table 1). Growth hormone probably influences CoQ_10_ consumption, due to accelerated metabolism, as in the case of hyperthyroidism.

## 6. Conclusions

Antioxidant systems represent a key defense mechanism in our body and an unbalance of these systems can underly a wide spectrum of human disease. The reported experimental data show that systemic hormones can affect their levels, both in physiological and pathological conditions. However CoQ10 can be affected by different mechanisms; its low levels in plasma, in fact, can be due to accelerated metabolism and/or consumption, such as in hyperthyroidism and acromegaly, or a reduced synthesis, such as in hypoadrenalism and hypogonadism (Figure 2).

All pituitary hormones and the dependent glands (thyroid, adrenal, gonads) are involved, even if an unequivocal picture is far from being designed. However, it seems clear that in all the considered conditions, the effect of thyroid hormones is predominant on other hormones in influencing CoQ_10_ plasma levels. Even though more controlled studies are needed, the clinical usefulness of CoQ_10_ determination for a diagnosis refinement, or CoQ_10_ oral supplementation as a support to the specific endocrine therapy, has already been demonstrated in many cases.

## Figures and Tables

**Table 1:** Effect of thyroid function and other hormonal disorders, alone and combined with hypothyroidism, on CoQ_10_ plasma levels (values are mean ± SEM).

	**Patients** (No)	**CoQ_10_** (μg/mL)	**CoQ_10_/Chol.** (nmol/mmol)	**Ref**.
**Control subjects**	21	0.68 ± 0.04	217.2 ± 20.3	32
**Hyperthyroidism**	25	0.45 ± 0.03	167.0 ± 20.5	
**Hypothyroidism**	27	1.04 ± 0.07	211.1 ± 11.2	
	
**Hypoadrenalism**	19	0.67 ± 0.06	188.1 ± 10.2	39
**Hypoadrenalism & Hypothyroidism**	19	0.92 ± 0.07	231.6 ± 32.4	
	
**Hypogonadism**	10	0.66 ± 0.06	190.8 ± 13.0	40
**Hypogonadism & Hypothyroidism**	6	1.11 ± 0.02	216.0 ± 25.0	
	
**Acromegaly**	10	0.50 ± 0.02	122.6 ± 11.6	41
**Acromegaly & Hypothyroidism**	4	0.98 ± 0.05	202.2 ± 15.3	
